# Poverty and Community-Acquired Antimicrobial Resistance with Extended-Spectrum β-Lactamase–Producing Organisms, Hyderabad, India

**DOI:** 10.3201/eid2408.171030

**Published:** 2018-08

**Authors:** Marcella Alsan, Nagamani Kammili, Jyothi Lakshmi, Anlu Xing, Afia Khan, Manisha Rani, Prasanthi Kolli, David A. Relman, Douglas K. Owens

**Affiliations:** National Bureau of Economic Research, Cambridge,; Massachusetts, USA (M. Alsan); Stanford University, Stanford, California, USA (M. Alsan, A. Xing, D.A. Relman, D.K. Owens);; Veterans Affairs Palo Alto Health Care System, Palo Alto, California, USA (M. Alsan, A. Xing, D.A. Relman, D.K. Owens);; Gandhi Medical College and Hospital, Secunderabad, India (N. Kammili, J. Lakshmi, M. Rani);; University of Chicago, Chicago, Illinois, USA (A. Khan);; Guntur Medical College and Hospital, Guntur, India (P. Kolli)

**Keywords:** antimicrobial resistance, community-acquired antimicrobial resistance, poverty, environment, extended-spectrum beta-lactamase, bacteria, India, ESBL, AMR

## Abstract

The decreasing effectiveness of antimicrobial agents is a global public health threat, yet risk factors for community-acquired antimicrobial resistance (CA-AMR) in low-income settings have not been clearly elucidated. Our aim was to identify risk factors for CA-AMR with extended-spectrum β-lactamase (ESBL)–producing organisms among urban-dwelling women in India. We collected microbiological and survey data in an observational study of primigravidae women in a public hospital in Hyderabad, India. We analyzed the data using multivariate logistic and linear regression and found that 7% of 1,836 women had bacteriuria; 48% of isolates were ESBL-producing organisms. Women in the bottom 50th percentile of income distribution were more likely to have bacteriuria (adjusted odds ratio 1.44, 95% CI 0.99–2.10) and significantly more likely to have bacteriuria with ESBL-producing organisms (adjusted odds ratio 2.04, 95% CI 1.17–3.54). Nonparametric analyses demonstrated a negative relationship between the prevalence of ESBL and income.

Antimicrobial resistance (AMR) is a growing global public health threat that could reverse decades of progress in increasing longevity around the world (*1*). The rapid pace of AMR spread coupled with a shortage of novel antimicrobial agents has led the World Health Organization (WHO) to warn of a “postantimicrobial era” (*2*). By 2050, deaths attributable to AMR could exceed 10 million per year, more than the number of deaths from cancer and traffic accidents combined, while costing the global economy US $60–$100 trillion of economic output (*3*). Most policy recommendations for curtailing AMR are tailored to wealthy nations; however, the threat of AMR is also severe for poorer countries (*4*,*5*). Infectious diseases are still common causes of illness and death in such settings, and the availability of second- or third-line therapies is limited (*6*,*7*). Yet, detailed estimates of the prevalence of AMR in developing countries are limited and often rely on samples from returning travelers or in-country patients receiving care for suspected infectious diseases. These data point to an alarming rate of community-acquired AMR (CA-AMR) in extended-spectrum β-lactamase (ESBL)–producing organisms, including in Hyderabad, India, the setting of this study (*8*–*11*). Such trends are of international concern given the possibility for AMR pathogens and genetic elements to spread across political and geographic boundaries. 

Because of the lack of detailed data from developing countries, empirical work on predictors of CA-AMR is generally limited to responses from wealthy countries or cross-country comparisons. These studies generally document robust positive relationships between antimicrobial drug use, healthcare provider contacts, and the prevalence of AMR (*12*–*15*). These strong links have led to the hypothesis that growing drug resistance in developing countries is attributable to rising incomes, which increase demand for many health products, including antimicrobial drugs (*16*–*18*). Yet, other factors that place the poor at greater risk for CA-AMR might be more pronounced in developing countries. First, the poor are more likely to be exposed to infectious agents from other humans and are at higher risk for illness because of malnutrition and immunodeficiency (*4*). Second, the poor are more likely to experience subinhibitory doses of antimicrobial agents because of shorter courses of treatment, sharing medication, or expired or low-quality drugs (*19*–*22*). Third, the poor may be more likely to acquire resistant pathogens or AMR genetic elements in their food or water, leading to CA-AMR (*23*–*25*). 

To clarify the prevalence of and risk factors for CA-AMR and the specific relationship with poverty for individual persons in a developing country context, we prospectively collected data on 1,836 primigravidae women in a large public hospital in Hyderabad, India, over 12 months. To reduce the probability that AMR was acquired through a healthcare contact or activity, our study population consisted of women who were carrying a pregnancy to term for the first time and had never been hospitalized for a pregnancy. We focused on ESBL-producing organisms because findings by other researchers demonstrated rising rates of community-acquired infections associated with these organisms (*8*–*11*,*15*). We complemented our microbiological sample collection with a detailed survey on sociodemographic information and assessed AMR risk factors using the US Demographic and Health Surveys tool AMR Module for Population-Based Surveys (*26**,**27*).

## Methods

### Study Design

The design was a cross-sectional observational study of women in Hyderabad, India, carrying a pregnancy to full term for the first time. The study was approved by the Indian Council of Medical Research (ICMR), the Institutional Review Board of Gandhi Medical College and Hospital, and the Administrative Panel for the Protection of Human Subjects (Institutional Review Board) of Stanford University.

### Setting and Participants

We conducted the study at Gandhi Medical College and Hospital, a large public teaching hospital in Hyderabad that provides free healthcare for all. We surveyed first-time pregnant women seeking antenatal care from October 1, 2015, through September 29, 2016. We identified eligible patients from the outpatient clinic roster. Patients were deemed eligible if they were <40 years of age, pregnant for the first time, and had not been interviewed on a prior visit. After obtaining informed consent, a team member interviewed patients in a quiet research office. One woman with an incomplete survey was omitted. Results are robust to her inclusion.

### Data Sources/Measurement

We performed urine culture and bacterial identification using ChromID CPS3 agar and the VITEK-2 system (both from BioMerieux, Marcy l’Etoile, France). We performed antimicrobial susceptibility testing and interpretations, including ESBL screening using the VITEK-2 ESBL test (identification and antimicrobial susceptibility pattern), in accordance with guidelines from the Clinical and Laboratory Standards Institute (Technical Appendix). We diagnosed bacteriuria when there were >10^5^ CFU of a single bacterial strain per milliliter of urine or when >2 different colony types were present and 1 had a colony count of >10^5^ CFU/mL.

A trained onsite investigator conducted a structured interview at the time of the antenatal visit using a questionnaire based on the Demographic and Health Surveys tool AMR Module for Population-Based Surveys (*27*). We queried patients on their usual and current residence, occupation, husband’s occupation, household income, religion, caste, education level, dietary and hygiene practices, and recent nonvitamin tablet consumption. Many of the women did not understand the original question about antimicrobial drug use, so we used ingestion of any tablet other than a vitamin as an upper bound on recent antimicrobial drug ingestion. Study staff also directed women to use the clean-catch urine sample technique. We sent all laboratory test results to the patient’s healthcare provider for further action. Definitions of variables included in the analysis are provided in the online Technical Appendix. 

### Statistical Methods

We performed 3 analyses to identify predictors of bacteriuria or growth of an ESBL-producing organism. First, we assessed univariate relationships among sociodemographic, clinical, and environmental exposures and bacteriuria or CA-AMR caused by ESBL-producing organisms; we used *t*-tests for continuous outcomes and χ^2^ tests for categorical outcomes.

Guided by the results from an unadjusted analysis, which demonstrated poverty as a significant predictor of the outcome, we used logistic regression to estimate the association between income and bacteriuria, as well as between income and bacteriuria from ESBL-producing organisms. The model also included background characteristics that might influence bacteriuria or AMR, such as respondents’ education level. We included age and Hindu religion because results for both were significant in 1 of the 2 univariate analyses. We included prior hospitalization and history of abortion because these variables reflect prior exposure to the inpatient medical system, which has been shown to be a risk factor for AMR in industrialized countries (*28*). We did not include previous antimicrobial drug use in our main model, even though it is included in a related analysis (Technical Appendix Figure 1). Because few study participants reported any tablet ingestion in the last 30 days, the confidence intervals were very large (*13*). The reported results on income were not sensitive to the inclusion of tablet ingestion.

Next, we used linear regression to assess the nonparametric relationship between income and CA-AMR from ESBL-producing organisms. Specifically, we divided income into quartiles and included these quartiles in the linear regression along with the covariates previously described and used in the logit regression. For the 59 respondents missing income information (3.2% of the sample), we generated predicted income values from husband’s occupation, education level, age at marriage, religion, and season. We normalized income per 10,000 rupees for graphing purposes. We performed the same analysis with the missing income values dropped rather than substituted with predicted values (Technical Appendix Figure 2).

## Results

The average age of women in the study was 21.8 years, slightly higher than the national average age at the time of first birth, which is 19.9 years (Table 1). Approximately one third (631/1,836) of the women surveyed were anemic; 862 (46.5%) were underweight and 288 (15.6%) reported a previous abortion, although none had previously carried a pregnancy to term. Forty-seven (2.6%) reported taking any nonvitamin tablet in the last 30 days.

**Table 1:** Unadjusted relationship between significant bacteriuria and sociodemographic, clinical, and environmental characteristics for pregnant women in Hyderabad, India*

Characteristic	All respondents, N = 1,836	>10^5^ CFU/mL bacteria in urine, n = 126	No bacteria in urine, n = 1,710	p value
Sociodemographic characteristics				
Mean age, y (SD)	21.81 (2.95)	22.05 (3.02)	21.79 (2.94)	0.35
Mean age at marriage, y (SD)	20.31 (2.87)	20.29 (3.22)	20.31 (2.84)	0.93
Low income, no. (%)†	932 (50.8)	75 (60.0)	857 (50.2)	**0.04**
Less than secondary education, no. (%)‡	322 (17.5)	23 (18.4)	299 (17.5)	0.81
Hindu, no. (%)§	1,228 (66.9)	72 (57.6)	1,156 (67.8)	**0.02**
Clinical characteristics, no. (%)				
Anemia	631 (33.6)	48 (38.4)	583 (34.2)	0.38
Low weight of mother	862 (46.5)	61 (48.8)	801 (47.0)	0.78
Previous abortion	288 (15.6)	18 (14.4)	270 (15.8)	0.80
Previous hospitalization¶	159 (8.7)	13 (10.4)	146 (8.6)	0.51
Tablet during last 30 days#	47 (2.6)	5 (4.0)	42 (2.5)	0.25
Dysuria**	220 (12.0)	16 (12.8)	204 (12.0)	0.78
Fever	84 (4.6)	5 (4.0)	79 (4.6)	1.00
Environmental and hygiene-related characteristics, no. (%)				
Household does not treat water	1,339 (72.9)	93 (74.4)	1,246 (73.0)	0.92
Household sewage not piped	90 (4.9)	8 (6.4)	82 (4.8)	0.39
Respondent strictly vegetarian	142 (7.7)	8 (6.4)	134 (7.9)	0.73
Handwashing <5 times/d	345 (18.8)	26 (20.8)	319 (18.7)	0.64

*Bold indicates statistical significance. p values were derived from *t*-test (for mean age variables) or χ^2^ test (for categorical variables). CFU, colony-forming units. †Income is total household income in previous 30 d; low income is an indicator variable for below the 50th percentile of income.  ‡Less than secondary education is an indicator variable for whether the participant reported no schooling or primary-only schooling. §Hindu is an indicator variable for Hindu religion.  ¶Hospitalization in the previous 12 mo.  #Tablet means any tablet other than a vitamin taken by the respondent in the 30 d before interview. We used the term “tablet” because “antimicrobial” was unclear for many respondents.  **For dysuria, an answer of “don't know” was coded as 0 (no).

A total of 126 respondents had significant bacterial growth in their urine, defined as >10^5^ CFU/mL. Gram-negative rods accounted for 107/126 (85%) of the isolates, including *Escherichia coli* (n = 75), *Klebsiella* (n = 28), *Sphingomonas* (n = 2), *Enterobacter* (n = 1,) and *Citrobacter* (n = 1). The remaining 19 isolates were gram-positive organisms, including *Staphylococcus*, *Streptococcus*, and *Enterococcus*; 1 organism was unknown. Of the ESBL isolates, 82% were *E. coli* and 18% were *K. pneumoniae*. At this level of prevalence, the study had 80% power to detect differences of ≈0.10 in the proportion of categorical variables and ≈0.75 in continuous variables. 

Women with significant bacteriuria did not substantially differ from those without bacteriuria in terms of age, age at time of marriage, or educational background (Table 1). Prevalence of anemia, dysuria, fever, and low weight were also not statistically different between the 2 groups, nor were hospitalizations within the previous year or use of nonvitamin tablets over the previous 30 days. Hindu participants were less likely to have high levels of bacteriuria than were those from other religious groups (p = 0.02), although we did not find a significant relationship between ESBL and religion. Women with bacteriuria were significantly more likely to fall in the lower half of the income distribution: 60.0% of women with bacterial growth had household incomes below the sample median, compared with 50.2% of women with no bacterial growth (p = 0.04).

We compared women with ESBL-producing bacteria with women without these bacteria (Table 2). Similar to the findings regarding bacteriuria, the demographic and clinical characteristics of women with ESBL-producing bacteria were not substantially different from those of women without these bacteria. However, 66.7% of women with ESBL-producing bacteria had household income below the median versus 50.4% of women without these bacteria, a significant finding (p = 0.01).

**Table 2:** Unadjusted relationship between community-acquired antimicrobial drug resistance caused by ESBL-producing organisms and sociodemographic, clinical, and environmental characteristics for pregnant women in Hyderabad, India*

Characteristic	All respondents, N = 1,836	ESBL present, n = 60	No ESBL present, n = 1,776	p value
Sociodemographic characteristics				
Mean age, y (SD)	21.81 (2.95)	22.65 (3.48)	21.78 (2.93)	**0.06**
Mean age at marriage, y (SD)	20.31 (2.87)	20.57 (4.19)	20.30 (2.82)	0.63
Low income, no. (%)†	932 (50.8)	40 (66.7)	892 (50.4)	**0.01**
Less than secondary education, no. (%)‡	322 (17.5)	10 (16.7)	312 (17.6)	1.00
Hindu, no. (%)§	1,228 (66.9)	36 (60.0)	1,192 (67.3)	0.27
Clinical characteristics, no. (%)				
Anemia	631 (33.6)	19 (31.7)	612 (34.6)	0.78
Low weight of mother	862 (46.5)	32 (53.3)	830 (46.9)	0.36
Previous abortion	288 (15.6)	11 (18.3)	277 (15.6)	0.59
Previous hospitalization¶	159 (8.7)	6 (10.0)	153 (8.6)	0.64
Tablet during last 30 days#	47 (2.6)	3 (5.0)	44 (2.5)	0.20
Dysuria**	220 (12.0)	7 (11.7)	213 (12.0)	1.00
Fever	84 (4.6)	2 (3.3)	82 (4.6)	1.00
Environmental and hygiene-related characteristics, no. (%)				
Household does not treat water	1,339 (72.9)	45 (75.0)	1,294 (73.1)	0.77
Household sewage not piped	90 (4.9)	4 (6.7)	86 (4.9)	0.53
Respondent strictly vegetarian	142 (7.7)	2 (3.3)	140 (7.9)	0.32
Handwashing <5 times/d	345 (18.8)	15 (25.0)	330 (18.6)	0.24

*p values were derived from *t*-test (for mean age variables) or χ^2^ test (for categorical variables). Bold indicates statistical significance. ESBL, extended-spectrum β-lactamase.  †Income is total household income in previous 30 d; low income is an indicator variable for below the 50th percentile of income. ‡Less than secondary education is an indicator variable for whether the participant reported no schooling or primary-only schooling. §Hindu is an indicator variable for Hindu religion. ¶Hospitalization in previous 12 mo. #Tablet means any tablet other than a vitamin taken by the respondent in the 30 d before interview. We used “tablet” because “antimicrobial” was unclear for many respondents. **For dysuria, an answer of “don't know” was coded as 0 (no).

**Figure 1:**
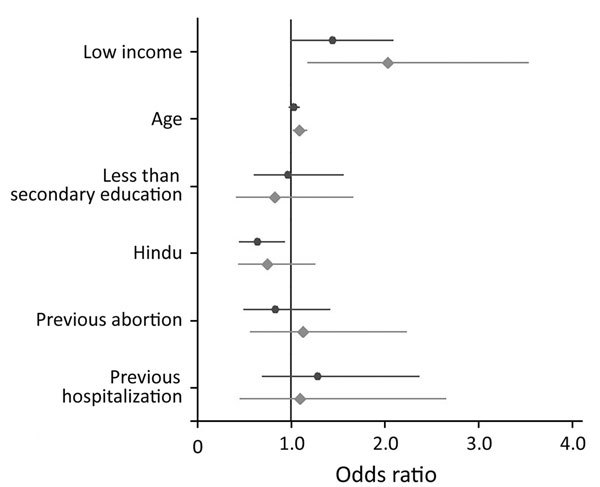
Adjusted odds ratios of bacteriuria and community-acquired antimicrobial resistance with ESBL-producing organisms by selected predictive variables for pregnant women in Hyderabad, India. Black dots represent odds ratios for bacterial growth in urine culture; lines indicate 95% CIs. Gray diamonds represent odds ratios for ESBL-producing organisms; lines indicate 95% CIs. The vertical line shows odds ratio = 1.0. ESBL, extended-spectrum β-lactamase.

Figure 1 shows the adjusted odds ratios (aOR) and 95% CIs for the relationships between respondent characteristics and the 2 study outcomes: any significant levels of bacteriuria or bacteriuria with ESBL-producing organisms. Being in the bottom half of the income distribution was associated with a greater likelihood of significant bacteriuria (aOR 1.44, 95% CI 0.99–2.10) and a greater likelihood of bacteriuria caused by ESBL-producing organisms (aOR 2.04, 95% CI 1.17–3.54). The only other predictor of ESBL that achieved statistical significance was age; higher age was positively associated with ESBL-producing organisms (aOR 1.09, 95% CI 1.02–1.18).

**Figure 2:**
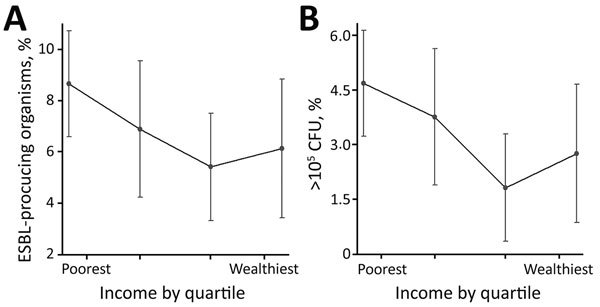
Nonparametric relationships between significant bacterial growth in urine culture and income (A) and between community-acquired antimicrobial resistance with ESBL and income (B) for pregnant women in Hyderabad, India, adjusted for respondent age, education level, income, religious background, hospitalization in previous 12 months, and previous abortion. Dots indicate adjusted mean predicted outcome; error bars indicate 95% CIs. Tick marks along baselines indicate quartiles of income. ESBL, extended-spectrum β-lactamase.

Nonparametric relationships between income and bacteriuria (Figure 2, panel A) and between income and ESBL (Figure 2, panel B), adjusted for the same set of covariates as in Figure 1, demonstrate a robust negative relationship between income quartile and both outcome variables. The poorest quartile had a predicted prevalence of 8.67% (95% CI 6.59%–10.73%) for significant bacteriuria and 4.68% (95% CI 3.23%–6.13%) for bacteriuria caused by ESBL organisms. When we restricted the analysis to women that who had substantial bacterial growth in the urine, we observed a similar ESBL–income gradient (Technical Appendix Figures 3, 4). This relationship did not occur between income and other potentially correlated outcome variables, such as hospitalization, past abortion, and tablet consumption (Technical Appendix Figure 5).

We estimated a model that included a first- and second-order term in income to investigate nonlinearities in the relationship between income and CA-AMR (Technical Appendix Figure 6). This approach was motivated by the observation of a slightly greater prevalence of ESBL bacteruria in the fourth versus the third income quartile (Figure 2, panel B). The coefficient on income was negative and significant (p = 0.001), consistent with the results from the other models. The coefficient on income squared was positive and significant (p<0.001), which implies a U-shaped function relating AMR and income. However, we calculated that the lowest ESBL risk occurs at an income of 24,000 rupees, which corresponds to roughly the 95th percentile of income in our sample. This finding implies that, for most women we surveyed, the probability of ESBL declines as household income rises.

## Discussion

This study produced 4 main results. First, 7% of women (126/1,836) had bacteriuria and ≈48% of their urine isolates were ESBL. This finding is striking for 2 reasons: almost none of these women reported recent antimicrobial drug use, and the sample (women carrying pregnancies to full term for the first time) was specifically chosen to mitigate healthcare-associated exposures that increase the risk for multidrug-resistant organisms. Second, women who had ESBL-producing organisms were less likely than those without these organisms to have been hospitalized recently or to report recent ingestion of nonvitamin tablets, thereby excluding some of the major correlates of AMR among wealthier populations. Third, the most robust predictor of whether women had clinically significant bacteriuria, including with ESBL-producing organisms, was household income. Outside of the top 5% of incomes, this relationship was negative and dose-responsive: the poorer the respondent, the higher her risk for CA-AMR. Fourth, if bacteriuria was present, income was still a robust predictor of ESBL, suggesting that the income–AMR gradient is not driven exclusively by living conditions that would place the poor at higher risk for any bacteriuria; rather, the poor are at higher risk specifically for CA-ESBL.

Theoretical arguments could be made for a link between higher income, antimicrobial consumption, and AMR. Yet, in the context of this study, when comparing across patients within a given public healthcare system that serves all regardless of their ability to pay, we found the opposite: a negative relationship between income and CA-AMR. There are several possible explanations for this finding. First, the poor might not be able to afford the highest quality antimicrobial drugs and may rely instead on expired pills or counterfeit brands, thereby increasing exposure to subinhibitory concentrations of antimicrobial drugs that fuel the emergence of drug-resistant strains (*29*). Second, impoverished persons are more likely to be poorly nourished and thus exposed to infectious diseases, increasing their demand for antimicrobial drugs relative to the wealthy. Given the low prevalence of nonvitamin tablet ingestion (an upper bound on use of oral antimicrobial drugs) in our data, however, these hypotheses seem less likely. Third, the higher prevalence of AMR among poorer persons could be due to contamination. This possibility also seems an unlikely explanation because the isolates we would consider to be members of the skin microbiota (e.g., staphylococcal and streptococcal species) were distributed evenly among the high- and low-income brackets. 

The most likely explanation, therefore, appears to be that the poor are exposed to an environmental source of antimicrobial drugs that is placing them at higher risk for CA-AMR than their wealthier peers, resulting in the CA-AMR wealth gradient that we observed. In Hyderabad, where we conducted this study, other researchers have noted levels of many antimicrobial drugs in wastewater treatment plants and treatment plant effluents, including ciprofloxacin, that are several-fold higher than maximal therapeutic plasma levels (*25*,*30*,*31*). 

Hindu religion was also marginally significant and protective in some of our specifications, but because our income measures are noisy, this variable may also be picking up relative socioeconomic status; in our sample, Hindu women reported higher mean incomes and higher education levels than women of other religious backgrounds. The differences seem unlikely to be related to diet because strict vegetarianism was not protective.

This study had some limitations. We gathered data at a single hospital in Hyderabad, India; results may differ in other impoverished communities with different environmental exposures and in wealthier populations. In addition, survey responses were self-reported and therefore subject to measurement error and surveyor demand bias; however, it is unclear how this fact might affect the relationship between income and AMR if women did not know their urine results when they answered the survey questions. Future research should attempt to verify some of the self-reported replies and use household consumption survey data in addition to estimates of income to measure poverty (*32*). Further, for reasons related to cultural sensitivity and logistical feasibility, we did not collect fecal samples, and such samples might have revealed different relationships than the urine samples did. In addition, because the study was performed at a large public hospital, we observed only the lower tail of the income distribution. Data from private hospitals that cater to the wealthy might show different patterns and, combined with our data from the public sector, might reveal a more robust U-shaped relationship between income and AMR in India. Finally, we identified 2 isolates of *Sphingomonas* spp., organisms commonly found in a variety of nonhost environments and occasionally identified as nosocomial pathogens (*2*) or as pathogens in pregnant women (*33*). One of the isolates was highly resistant to antimicrobial drugs, including all carbapenems in the study. The isolates did not have the same resistance pattern, however, so it is unlikely that these 2 cases were linked. 

Our study has several implications for policy. To date, recommendations for reducing CA-AMR often focus on reducing selective pressure for AMR emergence, including limiting outpatient antimicrobial prescriptions, in accordance with well-established research demonstrating the link between antimicrobial drug use and AMR (*34*,*35*). Our findings suggest that this recommendation may be insufficient when applied to the poorest of the poor in urban settings in developing countries. If the factors correlated with poverty, including environmental antimicrobial drug exposures, increase risk for AMR in these women, then a policy response should focus on identifying and mitigating such exposures. Future research should seek confirmation of our results in other community-dwelling populations, mapping potential hotspots of CA-AMR among the urban poor and identifying causative factors.

Technical Appendix Additional information about the relationships between poverty and antimicrobial resistance with extended-spectrum β-lactamase–producing organisms in pregnant women in Hyderabad, India.
