# Supplementary material for: Poverty and Community-Acquired Antimicrobial Resistance with Extended-Spectrum β-Lactamase–Producing Organisms, Hyderabad, India
Source: Emerg Infect Dis. 2018 Aug;24(8):1490–6. doi: 10.3201/eid2408.171030 (PMC6056104; doi:10.3201/eid2408.171030)
Supplement: Technical Appendix — Additional information about the relationships between poverty and antimicrobial resistance with extended-spectrum β-lactamase–producing organisms in pregnant women in Hyderabad, India. [file 17-1030-Techapp-s1.pdf]

Article DOI: https://doi.org/10.3201/eid2408.171030

Poverty and Community-Acquired Antimicrobial Resistance with Extended-Spectrum β-Lactamase–Producing Organisms, Hyderabad, India

Technical Appendix

ESBL Identification

Vitek 2 Compact is an automated system to rapidly identify organisms and assess their susceptibility pattern. The system uses an advanced expert system (AES) to interpret and validate the results. For ESBL, detection and production is based on the simultaneous assessment of the inhibitory effects of cefepime, cefotaxime and ceftazidime alone and in the presence of clavulanic acid. A Vitek card for susceptibility with a specific ESBL testing panel was inoculated and interpreted according to manufacturer recommendations (*1*,*2*).

Data and Variable Definitions

Sociodemographic Characteristics

Age

Self-reported age in years

Age Marriage

Self-reported age at marriage in years

Log Income

Log of self-estimated total household income in past 30 days, given in Indian Rupees and rounded

Low Income

Binary variable indicating woman's self-reported income falls in bottom half of distribution of reported incomes. Missing values are predicted using husband's occupation, age of marriage, religion, education level, and the month of the hospital visit.

Less than Secondary Education

Binary variable indicating woman reports highest level of schooling at primary or below

Hindu

Binary variable indicating woman's reported religion is Hindu

Clinical Characteristics

Anemia

Binary variable indicating woman reports that she currently has anemia

Low Weight Mother

Binary variable indicating woman reports that she is below weight

Previous Abortion

Binary variable indicating woman reports having had an abortion before this pregnancy

Previous Hospitalization

Binary variable indicating woman reports having been hospitalized $\geq$1 time in the past 12 months

Any Tablet During Last 30 days

Binary variable indicating woman reports taking a tablet for illness in the past 30 days, excluding iron pills, multivitamin, or calcium

Dysuria

Binary variable indicating whether woman reports she feels pain urinating

Fever

Binary variable indicating whether woman reports that she currently has fever

Environmental and Hygiene-Related Characteristics

Household Does Not Treat Water

Binary variable indicating woman reports her family does not treat its water to make it safer to drink

Household Sewage Not Piped

Binary variable indicating woman reports not having toilet facility connected to municipal sewerage system

Respondent Strictly Vegetarian

Binary variable indicating whether woman reports that she eats only vegetarian foods

Handwashing <5 times daily

Binary variable indicating whether woman answers how often she washes her hands with a value under 5 times daily

References

1. Singh RM, Singh HL. Comparative evaluation of six phenotypic methods for detecting extended-spectrum beta-lactamase-producing *Enterobacteriaceae*. J Infect Dev Ctries. 2014;8:408–15. PubMed http://dx.doi.org/10.3855/jidc.4052

Spanu T, Sanguinetti M, Tumbarello M, D'Inzeo T, Fiori B, Posteraro B, et al. Evaluation of the new VITEK 2 extended-spectrum beta-lactamase (ESBL) test for rapid detection of ESBL production in *Enterobacteriaceae* isolates. J Clin Microbiol. 2006;44:3257–62. PubMed http://dx.doi.org/10.1128/JCM.00433-06

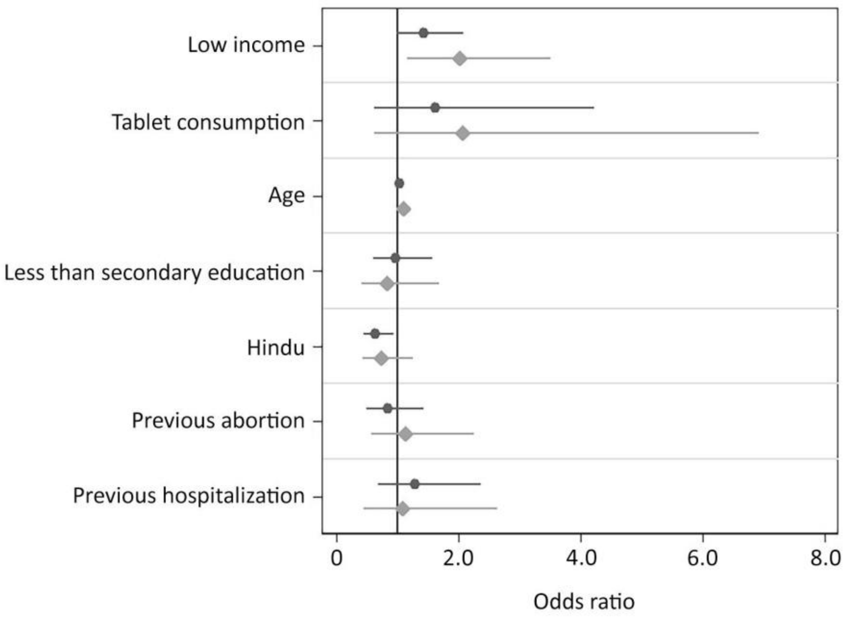

Technical Appendix Figure 1. Adjusted odds ratio of bacteriuria and community-acquired antimicrobial resistance with ESBL-producing organisms by selected predictive variables including tablet consumption. Black dots represent bacterial growth in urine culture; lines indicate 95% CIs. Gray diamonds represent ESBL-producing organisms; lines indicate 95% CIs. The vertical line shows odds ratio = 1.0.

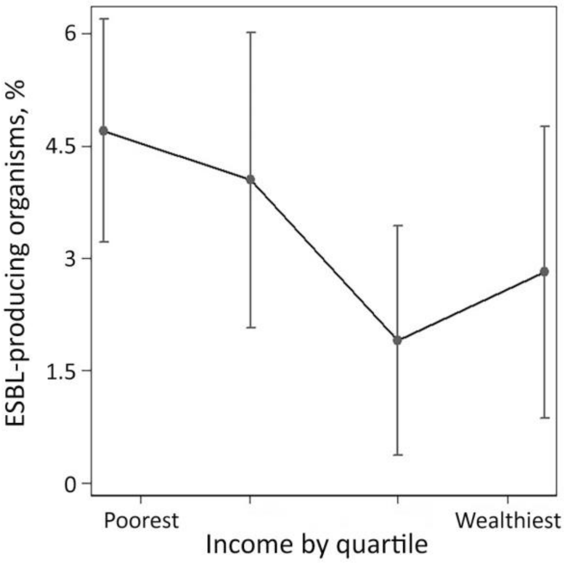

Technical Appendix Figure 2. Nonparametric relationship between income and bacteriuria with community-acquired antimicrobial resistance with ESBL-producing organisms. Figure shows the predicted mean outcome and 95% CI by income quartile adjusting for respondent age, education level, income, religious background, hospitalization in last 12 months, and previous abortion. Missing values for income (<5% of the sample) are not interpolated.

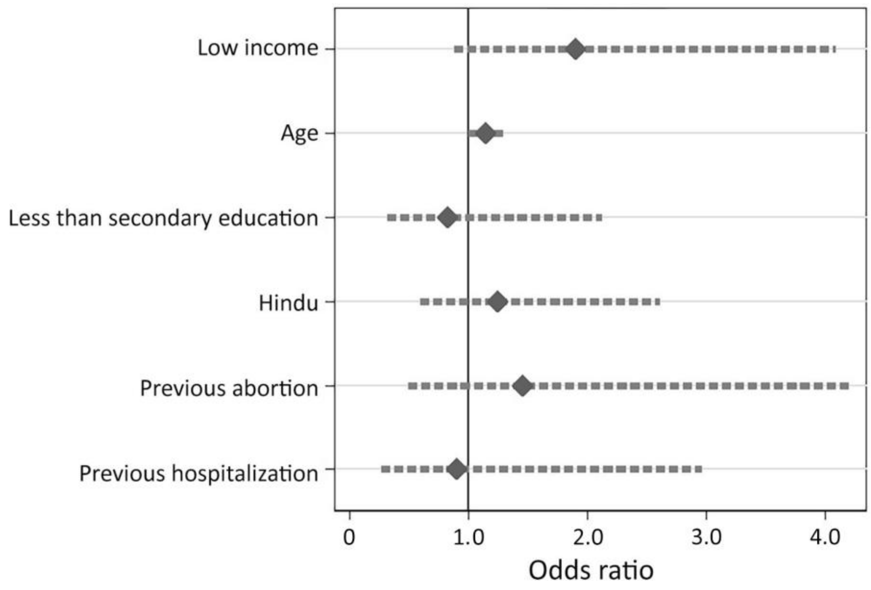

Technical Appendix Figure 3. Adjusted odds ratio of community-acquired antimicrobial resistance with ESBL-producing organisms among women with positive bacterial urinary culture growth. Diamonds represent odds ratios; lines indicate 95% CI. The vertical line shows odds ratio = 1.0.

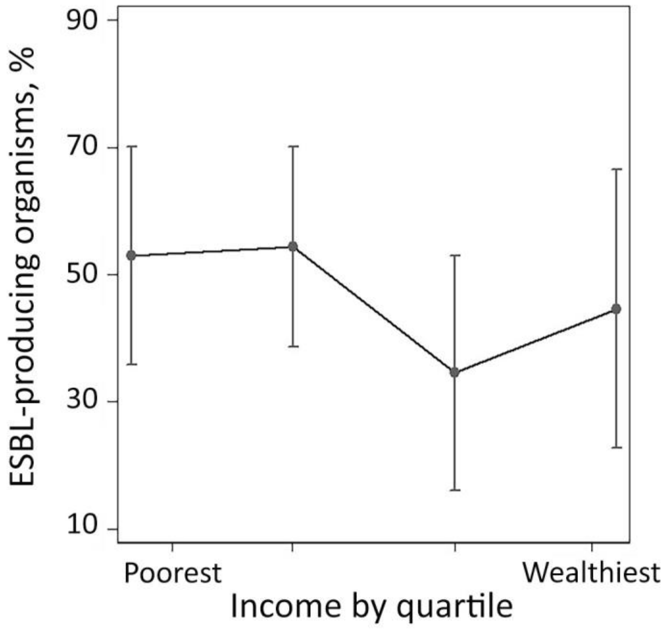

Technical Appendix Figure 4. Nonparametric relationship between community-acquired antimicrobial resistance with ESBL-producing organisms and income among women with positive bacterial urinary culture growth, adjusting for respondent's age, education level, income, religious background, whether they were hospitalized in the last 12 months and whether they had a history of abortion. Dots indicate adjusted mean predicted outcome by quartile of income and bars show 95% CI.

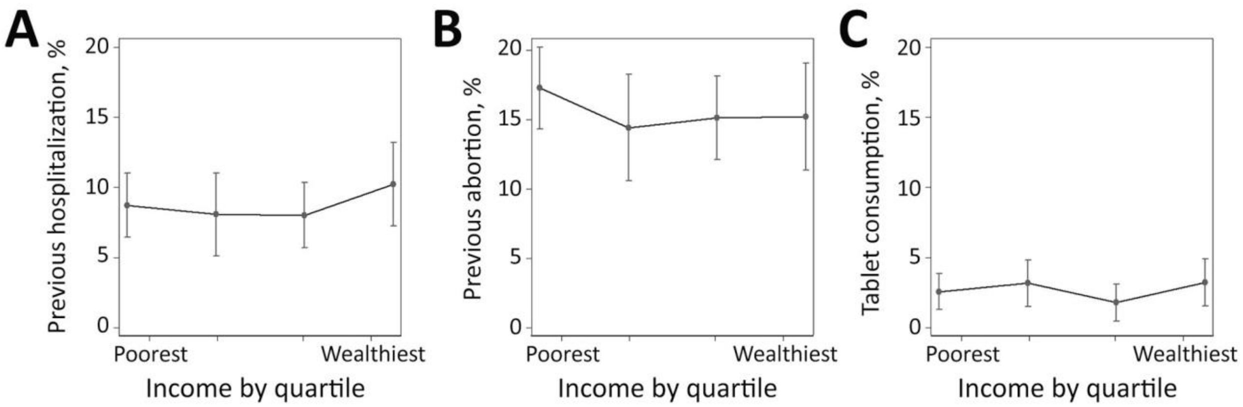

Technical Appendix Figure 5. Nonparametric relationships with patient household income. A) Relationship between hospitalization in the past year and income. Dots indicate the predicted percentage hospitalized by quartile of income and bars show 95% CI. B) Relationship between having an abortion before this pregnancy and income. Dots indicate predicted percentage of ≥1 previous abortion by quartile of income and bars show 95% CI. C) Relationship between tablet consumption in the past 30 days and income. Dots indicate predicted percentage consuming a tablet by quartile of income and bars show 95% CI.

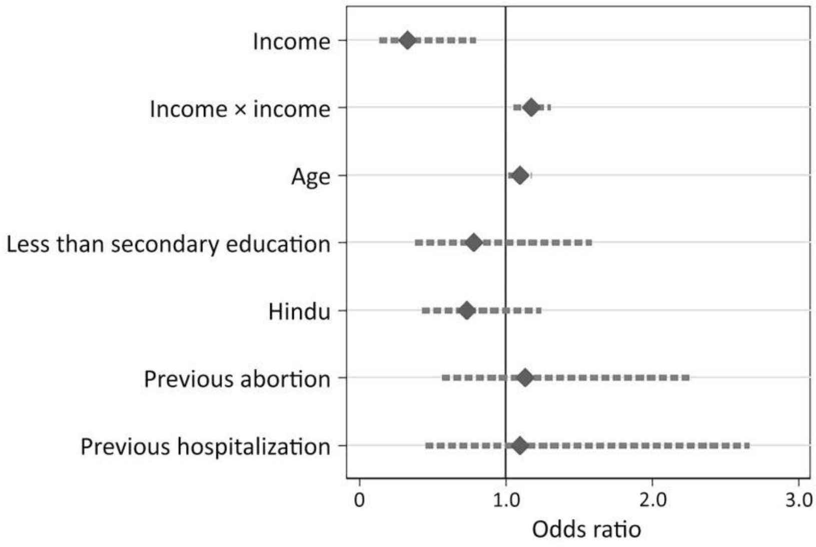

Technical Appendix Figure 6. Adjusted odds ratio and 95% CI for the relationship between significant bacterial urinary culture growth (black), and ESBL-producing organisms (gray) and various predictors. The vertical line indicates adjusted odds ratio = 1. The model includes a continuous linear and squared term in income (per 10,000 rupees); both are statistically significant and the latter is negative consistent with a U-shaped relationship between income and ESBL risk, although most study participants are at the part of the income distribution where the odds of ESBL are decreasing in income.
